# The mechanism of Zhenzhu Pills treating intracerebral hemorrhage secondary injury based on network pharmacology and molecular docking

**DOI:** 10.1097/MD.0000000000036837

**Published:** 2024-02-16

**Authors:** Gang Wu, Zeng Ren, Qingpei Hao, Yu Wong, Duo Zha, Xudong Cao, Ruen Liu

**Affiliations:** a Department of Neurosurgery, Peking University People’s Hospital, Beijing, P.R. China; b Department of Neurosurgery, People’s Hospital of Tibet Autonomous Region, Lhasa, Tibet Autonomous Region, P.R. China.

**Keywords:** components of traditional Chinese medicine, differentially expressed genes, intracerebral hemorrhage secondary injury, molecular docking, network pharmacology

## Abstract

**Background:**

Intracerebral hemorrhage (ICH) secondary injury is serious and affects patient’s prognosis. The Zhenzhu Pills used to treat subacute ICH in Tibet has shown to have a certain curative effect. Network pharmacology and molecular docking technology are employed to explore the potential mechanism of Zhenzhu Pills. The components and potential targets of Zhenzhu Pills were screened from the Traditional Chinese Medicine Systems Pharmacology database. The Gene Expression Omnibus Series 24265 was used to screen differentially expressed genes between perihematomal tissue and normal brain.

**Methods:**

The herbs–components–targets network was established, with weighted eigenvalue to identify the core components and targets of Zhenzhu Pills treatment of ICH secondary injury. Targets’ bioinformatics enrichment was proceeded by gene ontology and Kyoto Encyclopedia of Genes and Genome (KEGG) pathway analysis. Finally, molecular docking was used to identify the hydrogen bonding activity between the key components and action targets.

**Results:**

Five herbal drugs were screened from Traditional Chinese Medicine Systems Pharmacology database, with a total of 48 components and 234 targets. The Gene Expression Omnibus Series 24265 dataset was evaluated and 920 differentially expressed genes were identified. A total of 29 intersection targets of Zhenzhu Pills were explored in the treatment of ICH secondary injury. Drugs–components–targets network analysis showed that the pivotal targets were prostaglandin G/H synthase 2, interleukin 6, heme oxygenase-1, vascular endothelial growth factor, and vascular cell adhesion molecule 1, and the core components were quercetin, luteolin, and kaempferol. Gene ontology and KEGG pathway enrichment analysis showed that biological processes such as cell chemotaxis, wound healing, leukocyte migration, and regulation of body fluid levels played an important role in the secondary injury of ICH. The results of KEGG pathway analysis were mainly related to advanced glycation end products-receptor for advanced glycation end products signal pathway and tumor necrosis factor signal pathway. Molecular docking of 3 flavonoids with 5 core targets with the results also showed active hydrogen bonding.

**Conclusions:**

This study provides insights into the potential mechanisms of Zhenzhu Pills in the treatment of secondary injuries resulting from ICH and highlights specific components, targets, and molecular pathways involved in this therapeutic effect. These possible therapeutic mechanisms include inhibiting inflammation, edema, oxidative stress, and so on.

## 1. Introduction

The standardized incidence of intracerebral hemorrhage (ICH) in major Chinese cities is higher, ranging from 38.1 to 77.1 cases per 100,000 people per year, compared to developed countries like Europe and the United States, where it is lower.^[1–3]^ China also has the largest plateau area in the world, where high altitude and reduced oxygen levels significantly impact the cerebrovascular health of its residents. In fact, on the Tibetan Plateau, ICH accounts for a substantial 74.1% of all stroke cases, a much higher percentage than the global average of 6.3% to 41.3%.^[4]^ Patients with ICH in high-altitude areas face a twofold increase in disability risk compared to those at lower altitudes.^[5]^ Neurological dysfunction is believed to be linked to both the primary and secondary injuries associated with ICH. The primary injury of ICH occurs during the initial mechanical impact, where the sudden rupture of high-velocity blood flow from a perforator artery damages neuron fibers.^[6]^ The resulting high pressure is transmitted to the surrounding brain tissue, leading to hematoma-induced edema, ischemia, and herniation. The secondary injury, on the other hand, is a prolonged process characterized by the lysis of hematoma cells and the emergence of toxic metabolites.^[6]^ It could result in cytopathology changes surrounding hematoma, mitochondrial dysfunction, microglial cell activation, neurotransmitter disorders, and release of inflammatory factors. It is also suspected that this process may involve apoptosis, programmed cell death, and ferroptosis. Notably, the secondary injury associated with ICH is often more severe in high-altitude and hypoxic environments. Unfortunately, there are limited clinical treatment options for ICH’s secondary injury, with dehydration agents being one of the few available treatments.^[6]^

From 1981 to 2019, there have been 33.6% of food and drug administration-approved drugs derived from natural products or derivatives.^[7]^ Most of the traditional Chinese medicines have obvious advantages in treatment and safety, for thousands of years of application. In case of Zhenzhu Pills, prior studies have shown that their main components are effective in treating conditions such as ischemic stroke and subacute ICH in Tibet, with minimal observable toxic side effects.^[8,9]^ It has also been recorded in the Pharmacopoeia of the People’s Republic of China (2015 edition, Volume 1).^[10]^ The mechanism has been regarded as inhibiting inflammatory response,^[10]^ relieving lipid peroxidation, and downregulating the expression of caspase-3 in protecting the cerebral ischemia injury in vivo.^[11]^

**Figure 1.**
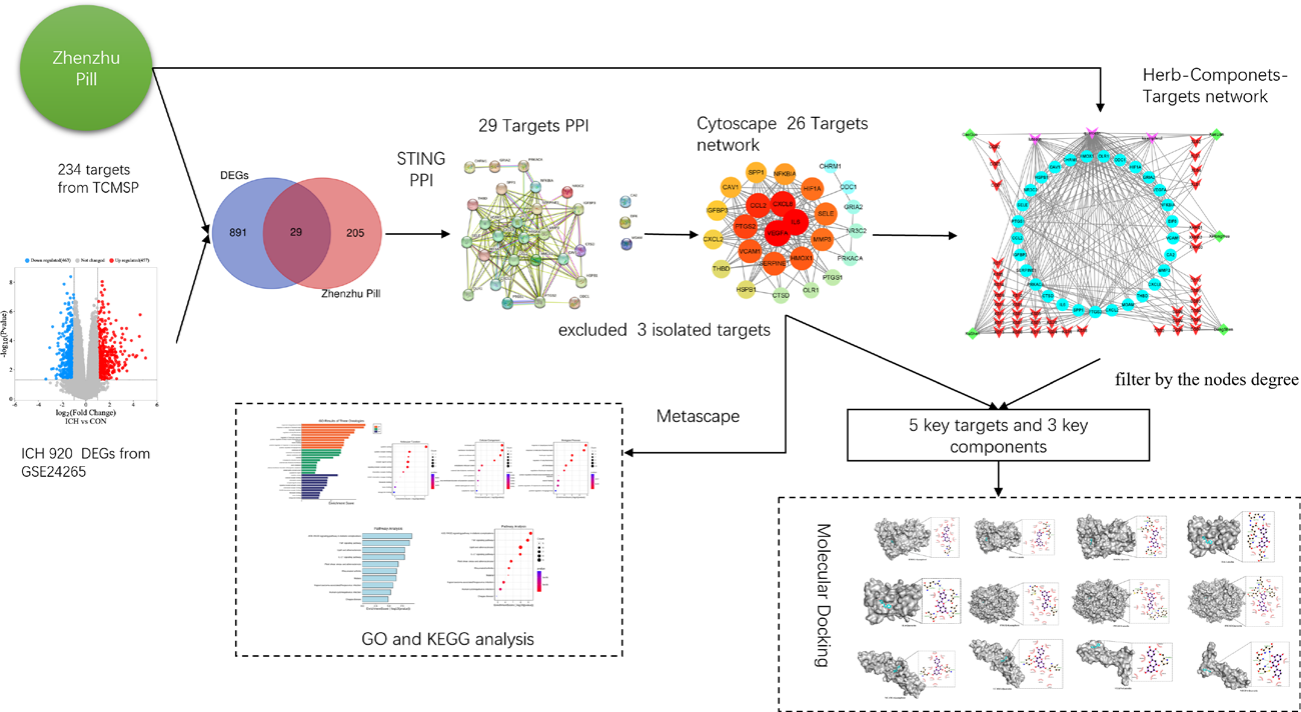
The approach of this manuscript and the flow chart.

Despite the long-standing use of traditional Chinese medicine, there has been a lack of systematic research on the pharmacological mechanisms of Zhenzhu Pills. To address this gap, the current study employs a network pharmacological approach to screen the main Chinese herbal medicine formulations, their chemical components, and potential targets within Zhenzhu Pills while investigating its possible pharmacological mechanisms. Furthermore, molecular docking^[12]^ is utilized to confirm the binding interactions between the components of Zhenzhu Pills and protein targets. The manuscript’s protocol and flowchart are outlined in Figure 1.

## 2. Materials and methods

### 2.1. Screening of components information of Zhenzhu Pills

The 5 main Chinese herbs (Caoguo, Xuelian, Dangshen, Kushen, and Xihonghua) in Zhenzhu Pills were screened from Traditional Chinese Medicine Systems Pharmacology (TCMSP) database (https://old.tcmsp-e.com/index.php).^[13]^ This data platform was organized into a network of 499 traditional Chinese medicines, 29,384 components, and 3311 disease-related gene targets, and indexes of pharmacokinetics parameters were established for screening the components. The properties of absorption, distribution, metabolism, and excretion were important characteristics of the components. Oral absorption availability (%) and drug likeness were important screening indexes. Potential active components were filtered by the criteria (oral bioavailability ≥ 30% and drug likeness ≥ 0.18) and saved in Smiles format.^[14]^ The study was approved by the Ethics Committee of Peking University People’s Hospital.

### 2.2. Predicting the targets of ICH secondary injury

The keywords “intracerebral hemorrhage,” “*Homo sapiens*,” and “brain samples” were retrieved in Gene Expression Omnibus (https://www.ncbi.nlm.nih.gov/gds) database, with the optimal result of Gene Expression Omnibus Series (GSE) 24265^[15]^ among the alternative 21 entries. GSE24265 were gathered with 11 specimens of 4 cases of ICH patients, including that of perihematomal tissue and contralateral white matter and gray matter. The ICH secondary injury-related gene can be detected by comparing the differentially expressed genes (DEGs) between perihematomal tissue and normal brain tissue. More than 45,000 transcripts were downloaded and normalizing by using R language, and DEGs of ICH were explored by limma package.^[16]^ The parameters of DEGs, fold change (|log2FC| > 1) and *P* value (<0.05), were the cutoff values.^[17]^

### 2.3. The intersection targets between Zhenzhu Pills active components and ICH secondary injury

The targets corresponding to Zhenzhu Pills components and ICH secondary injury DEGs were proceeded standardization by uniform names through UniProt (https://www.uniprot.org/) database.^[18]^ UniProt provides a set of regularly updated, high-quality, freely accessible protein functional information annotation and universal terms.^[19]^ The potential target genes of Zhenzhu Pills and the genes related to ICH secondary injury were imported into an online Venn diagram drawing website (http://bioinformatics.Psb.ugent.be/webtools/Venn/) to acquire the intersection genes.^[18]^

### 2.4. Construction of the protein–protein interaction network

The acquired intersection targets were imported into String database (https://string-db.org/),^[20]^ followed by the parameters being set as follows: species as “*Homo sapiens*,” the interaction threshold to middle confidence (>0.4), and other parameters as default value to obtain the protein–protein interaction (PPI) network. The results were imported into Cytoscape 3.9.1 (http://www.cytoscape.org/). The Network Analyzer plug-in was used to calculate the characteristics of the network topology. The degree, betweenness, and closeness of each node were calculated by the CytoHubba plug-in to explore and rank central elements of the networks.^[21]^

### 2.5. Construction of Zhenzhu Pills component and ICH secondary injury target network

The above interaction targets associated with Zhenzhu Pills components and formulary drugs were introduced into Cytoscape 3.9.1 software, as the nodes of the network of “drugs–components–targets,” respectively. Edges were used to connect single drugs with components and targets, and the relationship between these different levels was shown. The network topology analysis was also carried out by using the CytoHubba plug-in to predict and sort important components and targets, according to the weighted values. The core targets were obtained by sharing the top 10 targets with the PPI networks, according to the degree, betweenness, and closeness.

### 2.6. The targets annotation of biomolecular functions and signal pathways

Metascape (metascape.org) was utilized, a web-based portal, to obtain comprehensive genetic annotation and analysis resources.^[22]^ Gene ontology (GO) annotations of the potential targets and data related to Kyoto Encyclopedia of Genes and Genome (KEGG) were analyzed and gathered. GO analysis included 3 parts: biological process (BP), cellular component, and molecular function. GO and KEGG analysis showed that *P* value < 0.01 and false discovery rate < 0.05 were selected as the cutoff. The result of (−log *P* value) was the enrichment score evaluation index.^[23]^ Bioinformatics (https://www.bioinformatics.com.cn/), a free online platform, was applied for bioinformatics data analysis and visualization.^[24]^ GO annotation information and KEGG signal pathway enrichment results were plotted by the bioinformatics platform.

### 2.7. Core active component-target molecule docking

CB-Dock2 was an upgraded version of protein–ligand blind docking platform on web server, including cavity detection, docking, and homologous template fitting.^[25]^ The method predicted the binding site of the target protein depending on the curvature-based cavity detection approach^[26]^ and use of AutoDock Vina (version 1.1. 2)^[24]^ to predict and query the binding sites of ligands. The process mainly included the following steps. The core drug components’ molecular structures were download from TCMSP database with MOL2 format. Moreover, the crystals of key ICH targets were screened from Research Collaboratory for Structural Bioinformatics Protein Data Bank (http://www.rcsb.org/) database. The crystals model and corresponding ligands were successively imported into CB-Dock2, the binding sites were automatically identified, the center position and cavity size were calculated, and the docking box size was customized according to the queried ligands for docking. The three-dimensional model of molecular docking results were visualized by PyMol software (http://pymol.org),^[27]^ and the two-dimensional model and hydrogen bond were displayed by LigPlus.^[28]^

## 3. Results

### 3.1. Potential active components and related targets of Zhenzhu Pills in TCMSP database

The potential active components of 5 drugs in Zhenzhu Pills were screened from TCMSP, including 5 components of Caoguo, 15 components of Dangshen, 22 components of Kushen, 5 components of Xihonghua, and 7 components of Xuelian. After the target names and deduplication process were uniformed, 48 components of the 5 drugs’ formula and related 234 potential targets were extracted from raw data. (See Table S1, Supplemental Digital Content, http://links.lww.com/MD/L363, Table S2, Supplemental Digital Content, http://links.lww.com/MD/L366, and Table S3, Supplemental Digital Content, http://links.lww.com/MD/L373.)

### 3.2. The potential targets of ICH secondary injury and the PPI network

**Figure 2.**
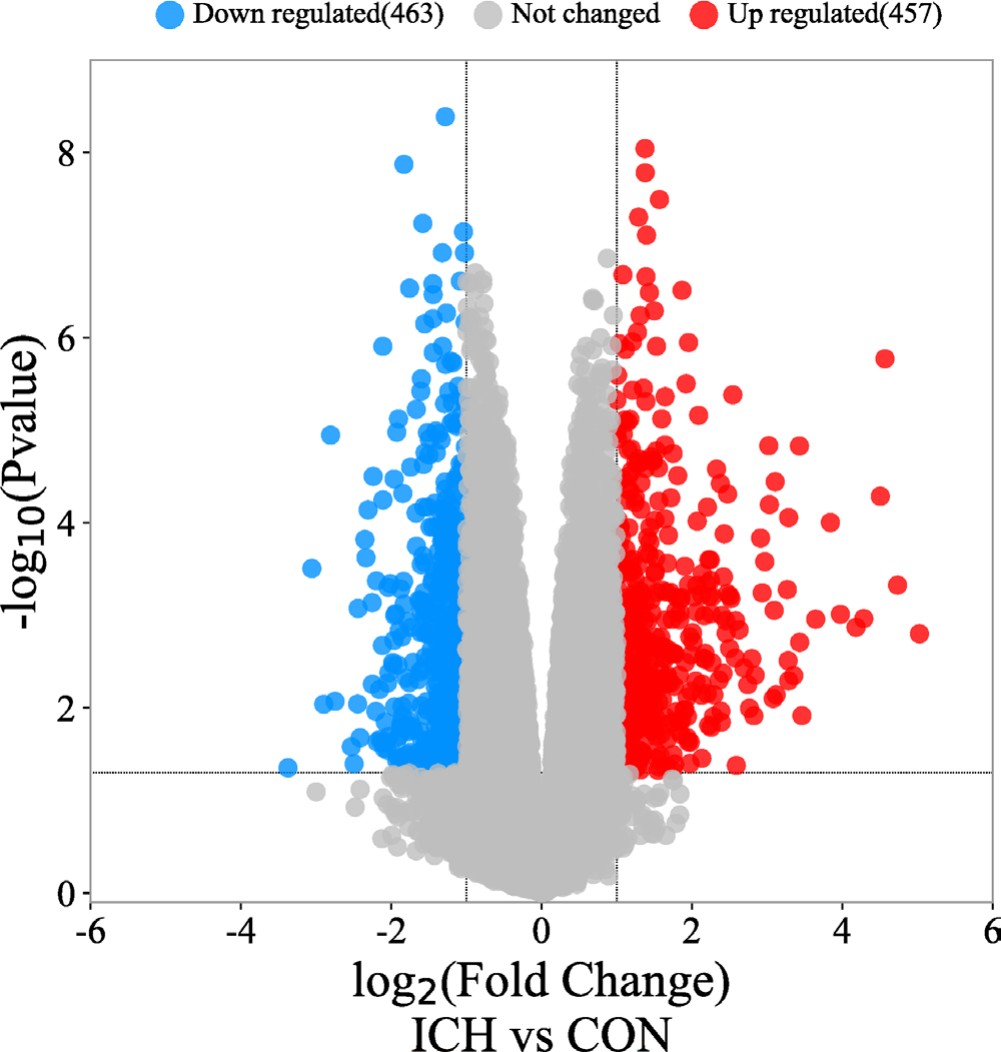
The volcano plot of DEGs of ICH secondary injury from GSE24265 calculated by limma package of R. There were 457 upregulated gene expressions and 463 downregulated gene expressions in ICH perihematomal area. ICH group was the intracerebral hemorrhage perihematomal area, and CON group was the contralateral white matter and gray matter. CON group = control group, DEGs = differentially expressed genes, GSE = Gene Expression Omnibus Series, ICH = intracerebral hemorrhage.

**Figure 3.**
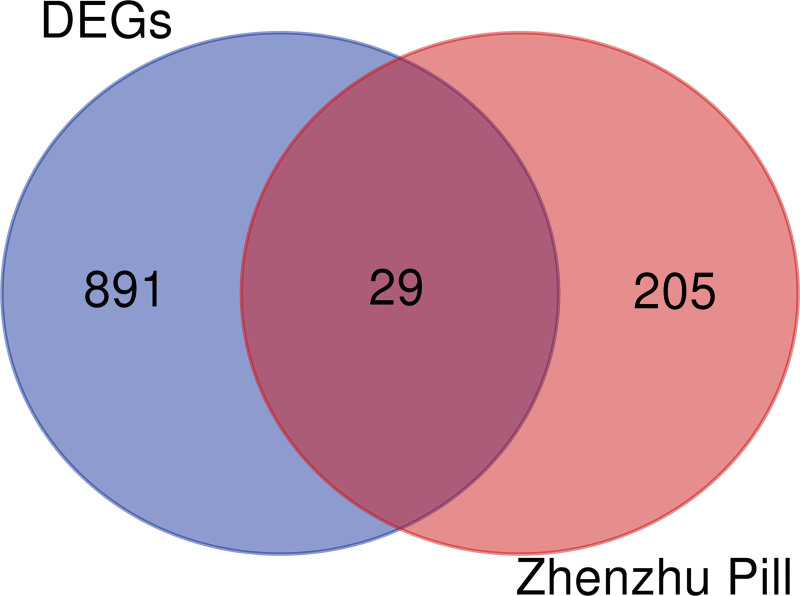
The intersection targets of Zhenzhu Pills screened from TCMSP and the DEGs of ICH secondary injury from GSE24264. DEGs = differentially expressed genes, GSE = Gene Expression Omnibus Series, ICH = intracerebral hemorrhage, TCMSP = Traditional Chinese Medicine Systems Pharmacology.

**Figure 4.**
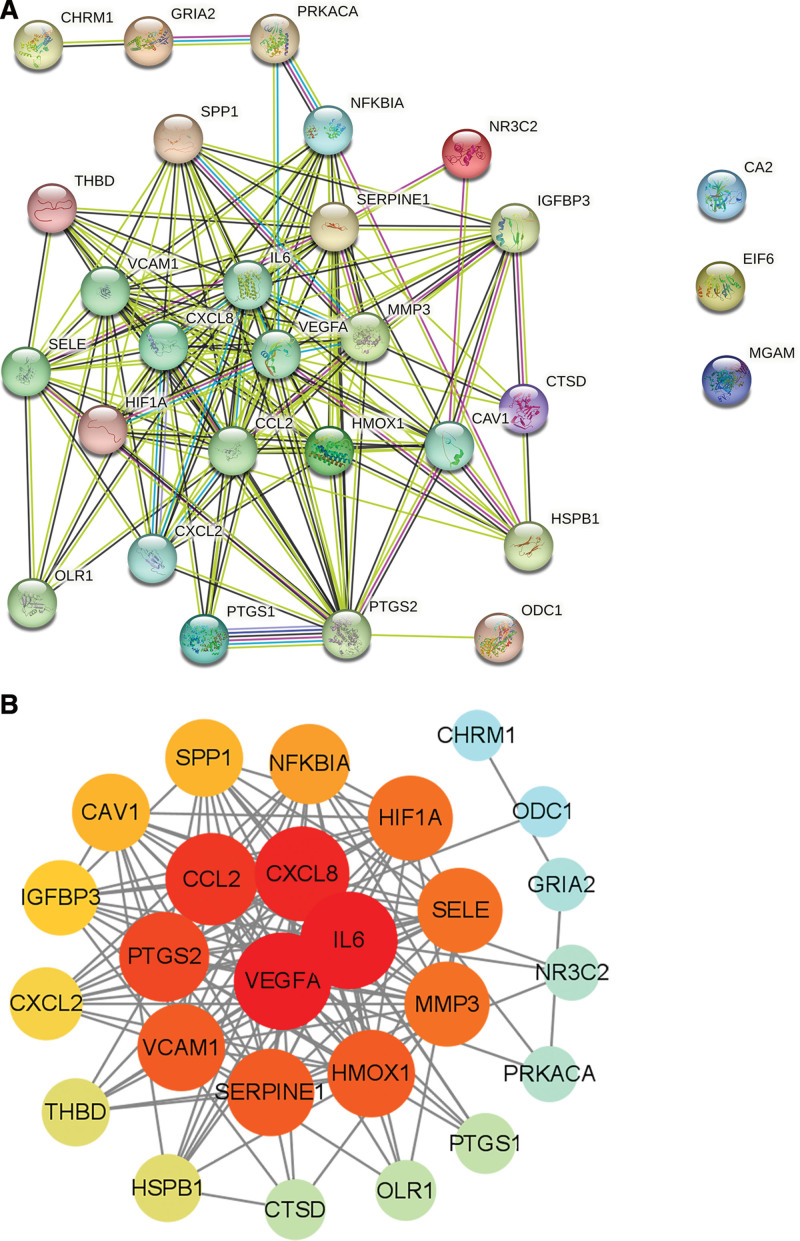
The PPI figure of the intersection targets of Zhenzhu Pills and ICH secondary injury. (A) The raw PPI figure from the String of 29 targets. Network nodes represent proteins. The edges of protein–protein connections are shown in different colors: light blue and purple edges represent known interactions (database and experiments); green, red, and blue edges represent predicted interactions (gene neighborhood, gene fusion, and gene cooccurrence); yellow edges represent text mining; black edges represent coexpression. (B) PPI data was re-sorted by Cytoscape software to exclude the 3 isolated proteins. The circle shape represented the targets, arranged in descending order according to degree value, from warm color to cool and large shape to small in spiral distribution. ICH = intracerebral hemorrhage, PPI = protein–protein interaction.

Comparing the gene expression between the surrounding tissues of cerebral hematoma and normal brain tissues, 20,487 disease-related targets were obtained from GSE24265 database (Table S4, Supplemental Digital Content, http://links.lww.com/MD/L374). After deduplication and deletion of nonhuman protein gene and uniform gene symbols through UniProt database, a total of 920 DEGs (463 genes downregulated and 457 genes upregulated) were obtained (Fig. 2; Table S5, Supplemental Digital Content, http://links.lww.com/MD/L375). There were 29 targets of active components obtained (Fig. 3). The 29 targets were imported into String database to calculate PPI correlation data, in which the edge was 143, the average node degree was 9.86, the average local clustering coefficient was 0.689, the expected number of edges was 30, and PPI enlargement *P* value was <1.0E-16 (Fig. 4A). After removing 3 isolated nodes, the PPI data were imported into Cytoscape 3.9.1 to calculate the network topology parameters and sorted by degree value (Fig. 4B). The results showed that the number of nodes was 26, number of edges was 143, and average number of neighbors was 11. The top 10 targets of degree value were vascular endothelial growth factor (VEGF), interleukin 6 (IL6), chemokine (C-X-C motif) ligand 8 (CXCL8), chemokine (C-C motif) ligand 2 (CCL2), prostaglandin G/H synthase 2 (PTGS2), serpin family E member 1 (SERPINE1), vascular cell adhesion molecule 1 (VCAM1), heme oxygenase-1 (HMOX1), gene matrix metalloproteinase-3 (MMP3), and Selectin E (SELE), respectively (Table S6, Supplemental Digital Content, http://links.lww.com/MD/L376).

### 3.3. Construction of the network of drugs active components and targets

**Figure 5.**
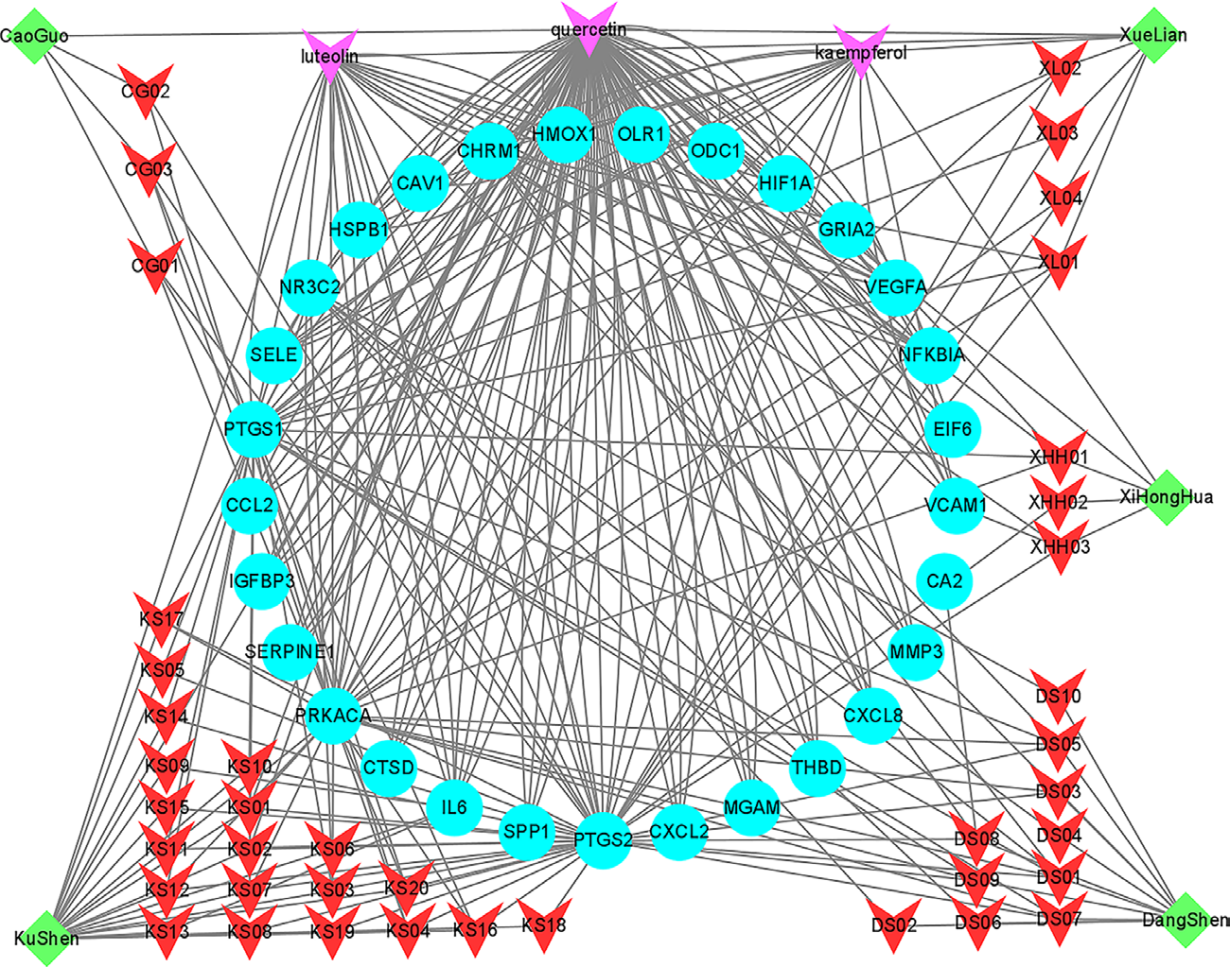
The network map constructed by herbs, active components, and the interaction targets of ICH secondary injury. Green diamond represented 5 kinds of traditional Chinese medicine names of Zhenzhu Pills, red arrow represents 41 kinds of unique active components, purple arrow represents multidrug coactive components (luteolin, quercetin, and kaempferol), and blue circle represents 26 potential therapeutic targets. ICH = intracerebral hemorrhage.

The information on 5 drugs, 44 active components, and 26 action targets were introduced into Cytoscape 3.9.1 with CytoHubba tool to calculate network parameters and the network of “drugs–active components–action targets” was constructed (Fig. 5). The network topology parameters were calculated by Network Analyzer with 77 nodes, 276 edges, and 4.753 as the average number of neighbors (Table S7, Supplemental Digital Content, http://links.lww.com/MD/L377). The top 3 active components were quercetin, luteolin, and kaempferol (Table 1), and the top 10 action targets were PTGS2, PTGS1, protein kinase CAMP-activated catalytic subunit alpha (PRKACA), IL6, cholinergic receptor muscarinic 1 (CHRM1), HMOX1, VEGF, VCAM1, nuclear factor of kappa light polypeptide gene enhancer in B-cells inhibitor, alpha (NFKBIA), and nuclear receptor subfamily 3, group C, member 2 (NR3C2) (Table S8, Supplemental Digital Content, http://links.lww.com/MD/L379). The core targets shared with the targets of ICH secondary injury network were PTGS2, IL6, HMOX1, VEGF, and VCAM1.

**Table 1:**
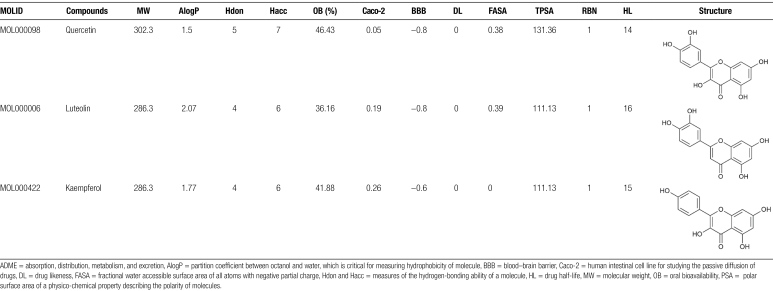
The ADME data and structure of the 3 core components.

### 3.4. Analysis of biological function and signal pathway

**Figure 6.**
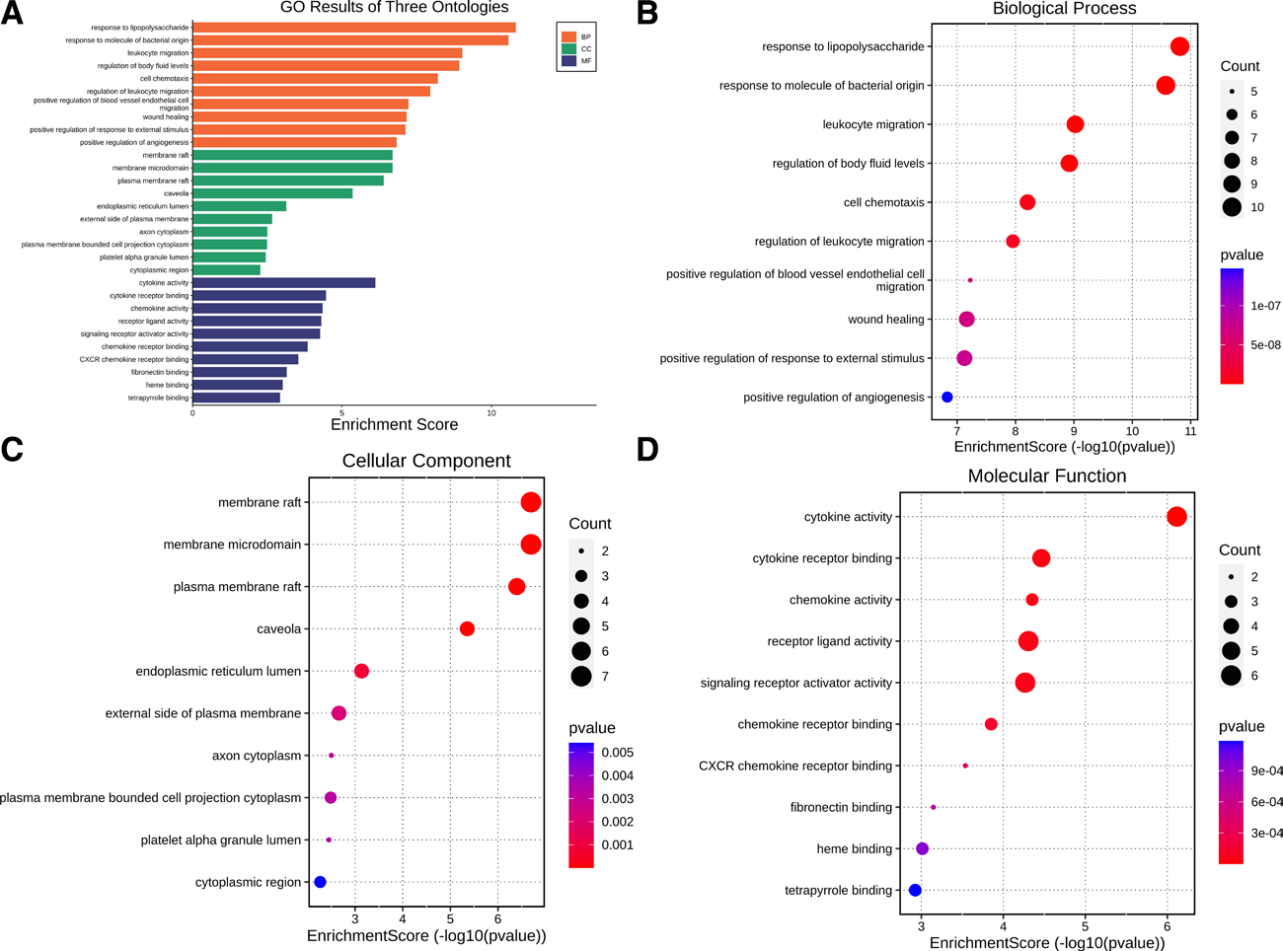
The biological function of ICH secondary injury by GO analysis. (A) Bar chart of 3 gene ontologies. (B) The bubble chart of biology process enrichment. (C) The bubble chart of cellular component enrichment. (D) The bubble chart of molecular function enrichment. GO = gene ontology, ICH = intracerebral hemorrhage.

**Figure 7.**
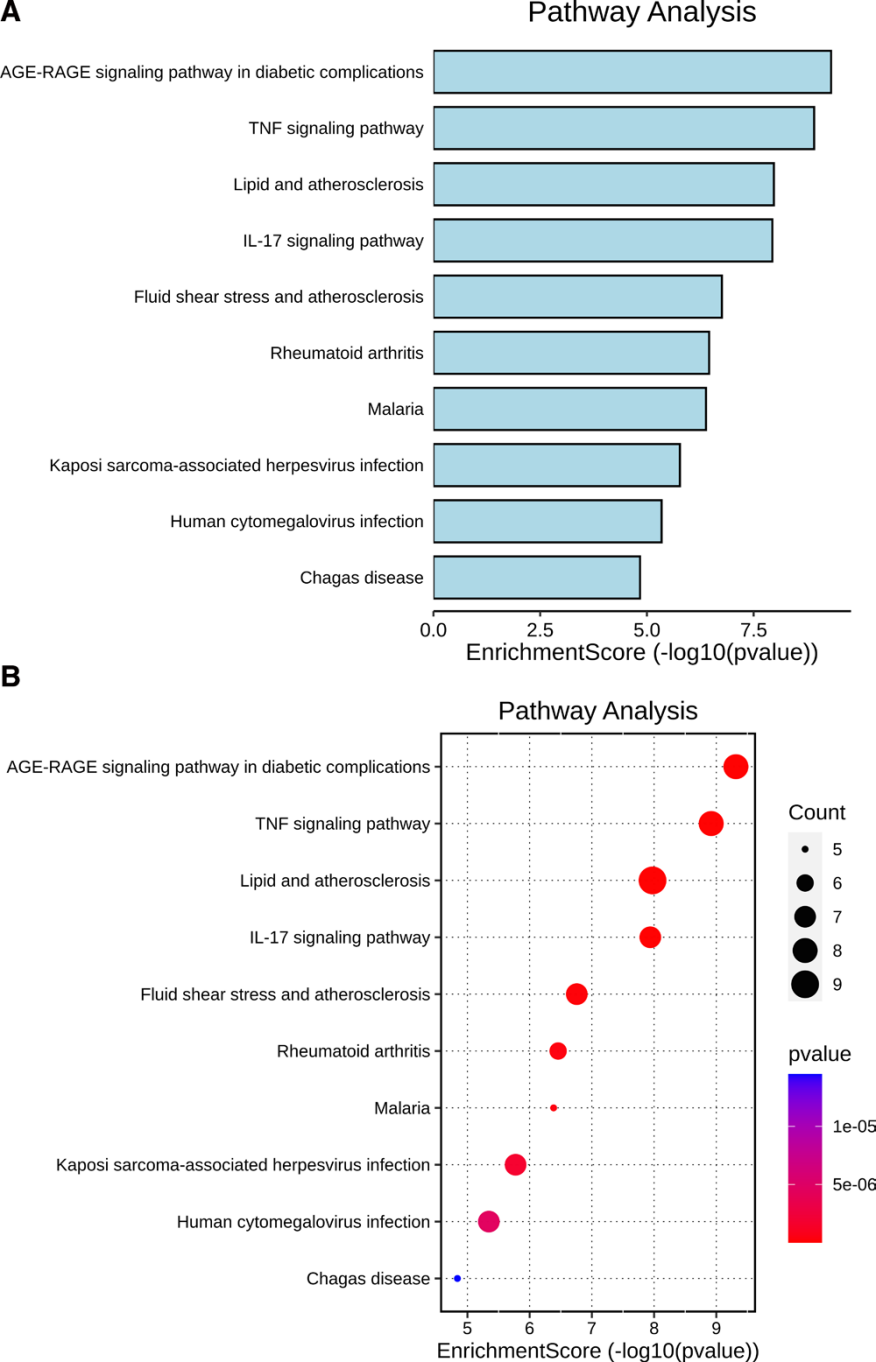
The pathway enrichment analysis by KEGG. (A) Bar chart of KEGG. (B) Bubble chart of KEGG. KEGG = Kyoto Encyclopedia of Genes and Genome.

The top 10 GO and KEGG analysis data were selected for display (Tables 2 and 3) and visualization (Figs. 6 and 7). From the BP level, Zhenzhu Pills had a greater impact on leukocyte migration (GO:0050900), regulation of body fluid levels (GO:0050878), cell chemotaxis (GO:0060326), regulation of leukocyte migration (GO:0002685), and wound healing (GO:0042060). From the cellular component level, Zhenzhu Pills had a greater impact on membrane raft (GO:0045121), membrane microdomain (GO:0098857), plasma membrane raft (GO:0044853), caveola (GO:0005901), and endoplasmic reticulum lumen (GO:0005788) in secondary injury. From the molecular function level, Zhenzhu Pills was related to cytokine activity (GO:0005125), cytokine receptor binding (GO:0005126), chemokine activity (GO:0008009), receptor ligand activity (GO:0048018), and so on. The first 10 pathways of KEGG are shown in Figure 7. It was known that Zhenzhu Pills might be involved in signal transduction pathways such as advanced glycation end products-receptor for advanced glycation end products (AGE-RAGE) signaling pathway (hsa04933), tumor necrosis factor (TNF) signaling pathway (hsa04668), lipid and atherosclerosis (hsa05417), IL17 signaling pathway (hsa04657), and fluid shear stress and atherosclerosis (hsa05418) in the treatment of ICH. The information about TNF signaling pathway related to ICH secondary injury is displayed in the Figure 9.

**Table 2:** The parameters and gene collection of the top 10 GO analysis.

ID	Description	GeneRatio	BgRatio	pvalue	p.adjust	qvalue	geneID	Count
Biological process (BP)								
GO:0032496	Response to lipopolysaccharide	10/26	343/18723	1.53E-11	2.4E-08	1.06E-08	CCL2/CXCL2/CXCL8/IL6/NFKBIA/PTGS2/SELE/SERPINE1/THBD/VCAM1	10
GO:0002237	Response to molecule of bacterial origin	10/26	363/18723	2.67E-11	2.4E-08	1.06E-08	CCL2/CXCL2/CXCL8/IL6/NFKBIA/PTGS2/SELE/SERPINE1/THBD/VCAM1	10
GO:0050900	Leukocyte migration	9/26	369/18723	9.46E-10	5.38E-07	2.38E-07	CCL2/CXCL2/CXCL8/HMOX1/IL6/SELE/SERPINE1/VCAM1/VEGFA	9
GO:0050878	Regulation of body fluid levels	9/26	379/18723	1.2E-09	5.38E-07	2.38E-07	CAV1/CHRM1/HIF1A/HSPB1/IL6/PRKACA/SERPINE1/THBD/VEGFA	9
GO:0060326	Cell chemotaxis	8/26	310/18723	6.22E-09	2.24E-06	9.89E-07	CCL2/CXCL2/CXCL8/HSPB1/IL6/SERPINE1/VCAM1/VEGFA	8
GO:0002685	Regulation of leukocyte migration	7/26	210/18723	1.11E-08	3.32E-06	1.47E-06	CCL2/CXCL8/HMOX1/IL6/SELE/SERPINE1/VEGFA	7
GO:0043536	Positive regulation of blood vessel endothelial cell migration	5/26	76/18723	5.93E-08	1.49E-05	6.6E-06	HIF1A/HMOX1/HSPB1/PTGS2/VEGFA	5
GO:0042060	Wound healing	8/26	422/18723	6.82E-08	1.49E-05	6.6E-06	CAV1/HIF1A/HMOX1/HSPB1/IL6/SERPINE1/THBD/VEGFA	8
GO:0032103	positive regulation of response to external stimulus	8/26	427/18723	7.47E-08	1.49E-05	6.6E-06	CXCL8/HSPB1/IL6/NFKBIA/PTGS2/SERPINE1/THBD/VEGFA	8
GO:0045766	Positive regulation of angiogenesis	6/26	181/18723	1.47E-07	2.41E-05	1.06E-05	CXCL8/HIF1A/HMOX1/HSPB1/SERPINE1/VEGFA	6
Cellular component (CC)								
ID	Description	GeneRatio	BgRatio	pvalue	p.adjust	qvalue	geneID	Count
GO:0045121	Membrane raft	7/26	335/19550	2.03E-07	1.22E-05	9.17E-06	CAV1/CTSD/HMOX1/OLR1/PRKACA/PTGS2/SELE	7
GO:0098857	Membrane microdomain	7/26	335/19550	2.03E-07	1.22E-05	9.17E-06	CAV1/CTSD/HMOX1/OLR1/PRKACA/PTGS2/SELE	7
GO:0044853	Plasma membrane raft	5/26	116/19550	4.02E-07	1.61E-05	1.21E-05	CAV1/HMOX1/PRKACA/PTGS2/SELE	5
GO:0005901	Caveola	4/26	84/19550	4.41E-06	0.000132	9.98E-05	CAV1/HMOX1/PTGS2/SELE	4
GO:0005788	Endoplasmic reticulum lumen	4/26	313/19550	0.00073	0.017509	0.013208	IGFBP3/IL6/PTGS2/SPP1	4
GO:0009897	External side of plasma membrane	4/26	421/19550	0.002177	0.043539	0.032845	GRIA2/SELE/THBD/VCAM1	4
GO:1904115	Axon cytoplasm	2/26	63/19550	0.00316	0.047545	0.035867	HIF1A/HSPB1	2
GO:0032838	Plasma membrane bounded cell projection cytoplasm	3/26	226/19550	0.003257	0.047545	0.035867	HIF1A/HSPB1/PRKACA	3
GO:0031093	Platelet alpha granule lumen	2/26	67/19550	0.003566	0.047545	0.035867	SERPINE1/VEGFA	2
GO:0099568	Cytoplasmic region	3/26	271/19550	0.00541	0.062353	0.047038	HIF1A/HSPB1/PRKACA	3
Molecular function (MF)								
ID	Description	GeneRatio	BgRatio	pvalue	p.adjust	qvalue	geneID	Count
GO:0005125	Cytokine activity	6/26	235/18368	7.65E-07	0.000117	7E-05	CCL2/CXCL2/CXCL8/IL6/SPP1/VEGFA	6
GO:0005126	Cytokine receptor binding	5/26	271/18368	3.44E-05	0.001654	0.00099	CCL2/CXCL2/CXCL8/IL6/VEGFA	5
GO:0008009	Chemokine activity	3/26	49/18368	4.44E-05	0.001654	0.00099	CCL2/CXCL2/CXCL8	3
GO:0048018	Receptor ligand activity	6/26	487/18368	4.94E-05	0.001654	0.00099	CCL2/CXCL2/CXCL8/IL6/SPP1/VEGFA	6
GO:0030546	Signaling receptor activator activity	6/26	495/18368	5.41E-05	0.001654	0.00099	CCL2/CXCL2/CXCL8/IL6/SPP1/VEGFA	6
GO:0042379	Chemokine receptor binding	3/26	72/18368	0.000141	0.003589	0.002148	CCL2/CXCL2/CXCL8	3
GO:0045236	CXCR chemokine receptor binding	2/26	18/18368	0.000291	0.006354	0.003803	CXCL2/CXCL8	2
GO:0001968	Fibronectin binding	2/26	28/18368	0.000712	0.013617	0.00815	IGFBP3/VEGFA	2
GO:0020037	Heme binding	3/26	139/18368	0.000971	0.0165	0.009876	HMOX1/PTGS1/PTGS2	3
GO:0046906	Tetrapyrrole binding	3/26	149/18368	0.001186	0.018148	0.010862	HMOX1/PTGS1/PTGS2	3

GO = gene ontology.

**Table 3:** The parameters and gene collection of the top 10 KEGG analysis.

ID	Description	GeneRatio	BgRatio	pvalue	p.adjust	qvalue	geneID	Count
hsa04933	AGE-RAGE signaling pathway in diabetic complications	8/26	100/8192	4.84E-10	8.57E-08	5.60E-08	CCL2/CXCL8/IL6/SELE/SERPINE1/THBD/VCAM1/VEGFA	8
hsa04668	TNF signaling pathway	8/26	112/8192	1.21E-09	1.07E-07	6.99E-08	CCL2/CXCL2/IL6/MMP3/NFKBIA/PTGS2/SELE/VCAM1	8
hsa05417	Lipid and atherosclerosis	9/26	215/8192	1.06E-08	5.09E-07	3.33E-07	CCL2/CXCL2/CXCL8/IL6/MMP3/NFKBIA/OLR1/SELE/VCAM1	9
hsa04657	IL17 signaling pathway	7/26	94/8192	1.15E-08	5.09E-07	3.33E-07	CCL2/CXCL2/CXCL8/IL6/MMP3/NFKBIA/PTGS2	7
hsa05418	Fluid shear stress and atherosclerosis	7/26	139/8192	1.75E-07	6.19E-06	4.05E-06	CAV1/CCL2/HMOX1/SELE/THBD/VCAM1/VEGFA	7
hsa05323	Rheumatoid arthritis	6/26	93/8192	3.49E-07	1.03E-05	6.73E-06	CCL2/CXCL2/CXCL8/IL6/MMP3/VEGFA	6
hsa05144	Malaria	5/26	50/8192	4.12E-07	1.04E-05	6.82E-06	CCL2/CXCL8/IL6/SELE/VCAM1	5
hsa05167	Kaposi sarcoma-associated herpesvirus infection	7/26	194/8192	1.68E-06	3.73E-05	2.44E-05	CXCL2/CXCL8/HIF1A/IL6/NFKBIA/PTGS2/VEGFA	7
hsa05163	Human cytomegalovirus infection	7/26	225/8192	4.53E-06	8.90E-05	5.82E-05	CCL2/CXCL8/IL6/NFKBIA/PRKACA/PTGS2/VEGFA	7
hsa05142	Chagas disease	5/26	102/8192	1.45E-05	0.00023	0.000153	CCL2/CXCL8/IL6/NFKBIA/SERPINE1	5

IL17 = interleukin 17, KEGG = Kyoto Encyclopedia of Genes and Genome, TNF = tumor necrosis factor.

**Figure 8.**
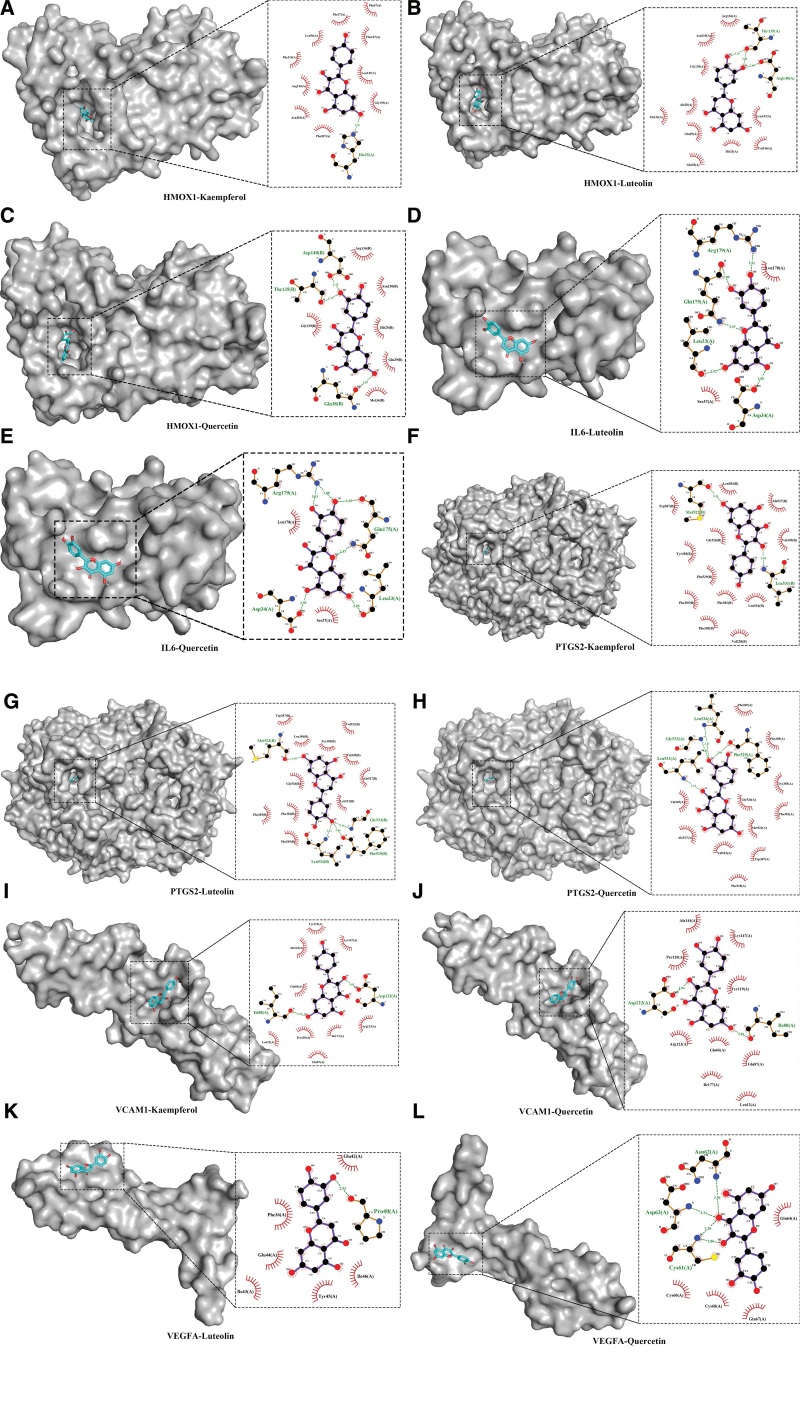
The three-dimension surface model and two-dimension model of molecular docking.

**Figure 9.**
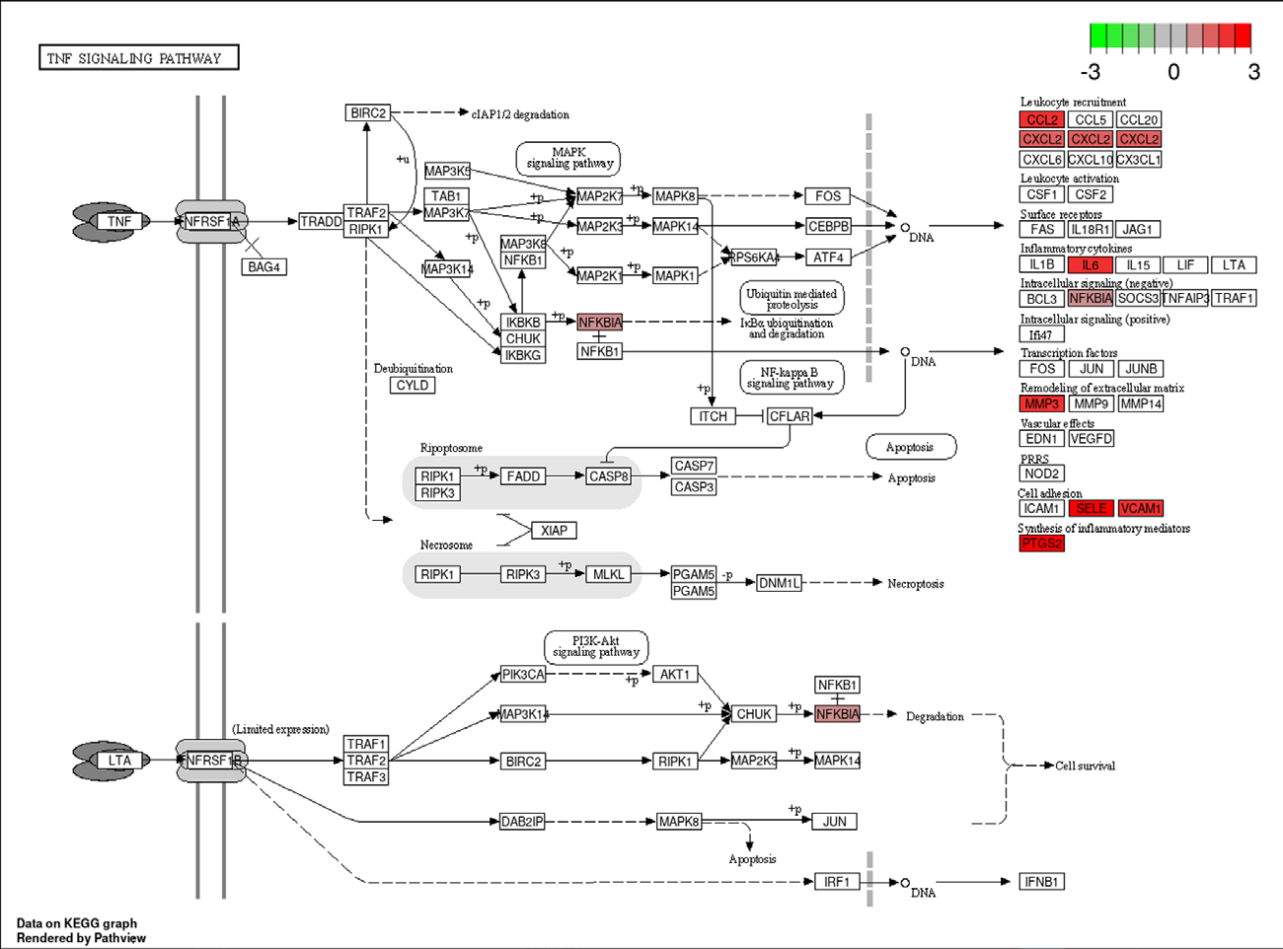
TNF signaling pathway. The red rectangle represents proteins upregulated from the intersection targets of Zhenzhu Pills and ICH secondary injury in the PPI network. ICH = intracerebral hemorrhage, PPI = protein–protein interaction, TNF = tumor necrosis factor.

### 3.5. Molecular docking

CB-Dock could simplify the docking process and improve the accuracy.^[25]^ The 3 core components in the active component–target–pathway network were docked with the 5 pivotal targets of ICH secondary injury. The molecular results are shown as the data list in Table 4 (Table S9, Supplemental Digital Content, http://links.lww.com/MD/L380) and as plot in Figure 8. Protein molecular residues and core components were bound with hydrogen bonds, respectively, HMOX1 and kaempferol binding residue as His25; HMOX1 and luteolin binding residues as Thr135 and Asp140; HMOX1 and quercetin binding residues as Gln38, Thr135, and Asp140; IL6 and luteolin binding residues as Asp34, Leu33, Gln175, and Arg179; IL6 and quercetin binding residues as Asp34, Leu33, Gln175, and Arg179; PTGS2 and kaempferol binding residues as Leu531 and Met522; PTGS2 and luteolin binding residues as Leu534, Phe529, Gly533, and Met522; PTGS2 and quercetin binding residues as Leu531, Gly533, Leu534, and Phe529; VCAM1 and kaempferol binding residues Ile88 and Asp122; VCAM1 and quercetin binding residues as Asp122 and Ile88; VEGFA and luteolin binding residue as Pro40; and VEGFA and quercetin binding residues as Cys61, Asp63, and Asn62.

**Table 4:** The parameter and VENA score of molecular docking between active components and core targets.

Key Targets	PDB ID	Components	Cavity volume	center_x	center_y	center_z	size_x	size_y	size_z	VENA score
PTGS2	5f19	Quercetin	4392	13.702	49.228	64.724	21	30	29	−9.4
		Luteolin	5179	11.893	52.644	16.694	33	21	29	−9.8
		Kaempferol	5179	11.893	52.644	16.694	33	21	29	−9.5
IL6	1alu	Quercetin	218	0.128	−23.19	−2.198	21	21	21	−6.3
		Luteolin	218	0.128	−23.19	−2.198	21	21	21	−6.7
HMOX1	1n45	Quercetin	1198	25.14	22.403	−33.049	21	21	28	−8.1
		Luteolin	1328	18.054	0.501	2.876	21	21	21	−8.1
		Kaempferol	1328	18.054	0.501	2.876	21	21	21	−7.5
VEGFA	4kzn	Quercetin	43	−14.053	0.872	3.48	21	21	21	−5.7
		Luteolin	43	−14.053	0.872	3.48	21	21	21	−5.8
VCAM1	1ij9	Quercetin	82	15.74	87.339	11.24	21	21	21	−7.1
		Kaempferol	82	15.74	87.339	11.24	21	21	21	−7

HMOX1 = heme oxygenase-1, IL6 = interleukin 6, PDB = Protein Data Bank, PTGS2 = prostaglandin G/H synthase 2, VCAM1 = vascular cell adhesion molecule 1, VEGFA = vascular endothelial growth factor A.

## 4. Discussion

The secondary brain injury typically manifests 2 to 14 days following the onset of ICH and is a primary contributor to the exacerbation of neurological dysfunction.^[29]^ This injury is underpinned by mechanisms such as programmed cell death, apoptosis, blood–brain barrier impairment, and increased brain edema, which are driven by pathological processes like oxidative stress and inflammation.^[3,30]^ It is important to develop complementary therapeutic method for secondary injury. Zhenzhu Pills, a traditional medical formula, has been used for treatment of subacute ICH in Tibet for years and thought to be anti-secondary injury. It is the challenging to explore the complex mechanism of Zhenzhu Pills in the plateau area with an altitude about 3700 meters. The results suggest that Zhenzhu Pills may safeguard the perihematomal brain from secondary damage by engaging in multiple BPs, including anti-inflammatory, antioxidative stress, and antiedema activities.

Utilizing network pharmacology, it has been discerned that quercetin, luteolin, and kaempferol are the primary pharmacological components within Zhenzhu Pills that combat ICH secondary injury. Their molecular structures are similar, belonging to flavonoids with polyphenol structures and C6–C3–C6 carbon skeleton, including 2 aromatic rings, connected with 3 carbon chains or oxygen-containing heterocycles (Table 1).^[31]^ They are found in fruits, vegetables, and traditional Chinese medicine and have a broad impact on inflammation and antioxidant processes.^[32]^ Notably, quercetin is known to protect neurons after ischemia-reperfusion injury^[33]^ and ameliorate neurological dysfunction in vivo with ICH.^[34]^ Luteolin can promote the activation of oxidative stress regulatory target protein Nrf2 and expression of downstream genes to protect traumatic brain injury in vivo and in vitro.^[35]^ Furthermore, it can mitigate the neuroinflammatory response following ICH by downregulating the activity of the TNF signaling pathway.^[36]^ Kaempferol could inhibit apoptosis induced by oxidation and inflammatory stress in rats with ischemia-reperfusion injury.^[37]^ Consequently, these 3 core components within Zhenzhu Pills may play a multipronged, multitarget role in protecting against ICH secondary injury.

Microglia and cerebral vascular endothelial cells activated by nervous system injury induce chemotaxis and inflammatory response.^[38]^ In the prodromal stage of inflammatory response, proinflammatory genes IL6^[39]^ and VCAM1^[40]^ are expressed and chemotaxis of inflammatory reactive cells. TNF and AGE-RAGE signal transduction are involved in the damage of blood–brain barrier and white matter fibers in ICH, also related to ferroptosis.^[41,42]^ As the inflammatory stage unfolds, glial cells, vascular endothelium, and neutrophils release reactive oxygen species (ROS) that harm adjacent tissues.^[38]^ These bioinformatics processes are also apparent in our research.

IL6 is a pivotal proinflammatory cytokine in the nervous system.^[39]^ Microdialysis studies have demonstrated significantly higher concentrations of IL6 in the perihematomal area compared to nonhematoma brain tissue within 20 to 26 hours following hematoma aspiration.^[43]^ The human GSE24265 dataset further supports this, revealing significantly higher DEGs of IL6 around the hematoma compared to control tissue (LogFC = 2.390 and *P* value = 0.014; Table S5, Supplemental Digital Content, http://links.lww.com/MD/L375). Notably, patients with elevated IL6 levels have a poorer prognosis in clinical trials such as factor seven for acute hemorrhagic stroke, a multicenter, randomized, double-blind, placebo-controlled 3-phase trial involving recombinant factor VIIa in the treatment of spontaneous ICH.^[44]^ Trozumab, an IL6-specific antibody, mitigates inflammation and reduces neuronal cell death in a rabbit model of subarachnoid hemorrhage.^[45]^ This study reveals the involvement of IL6 in multiple signaling pathways, including AGE-RAGE, TNF, lipid metabolism, atherosclerosis, and the IL17 signaling pathway, all of which are related to inflammatory responses.

VCAM1 is involved in leukocyte adhesion and aggregation.^[40]^ VCAM1 may be involved in neuronal apoptosis and pathophysiology in ICH rats.^[46]^ The neuronal injury is protected in rats, by downregulating the expression of VCAM1.^[40]^ Our simulated molecular docking results indicate that quercetin and luteolin exhibit strong molecular affinity with IL6, while quercetin and kaempferol display strong affinity with VCAM1, potentially inhibiting post-ICH inflammatory processes.

PTGS2, also known as cyclooxygenase-2 (COX2), is increased in the tissues around hematoma in experimental ICH, which may be related to secondary injury.^[47]^ This observation aligns with our findings in the GSE24265 dataset, which showed elevated PTGS2 expression (LogFC = 2.589 and *P* value = 0.042; Table S5, Supplemental Digital Content, http://links.lww.com/MD/L375). PTGS2 also might play a dual biological function in aggravating ICH secondary injury. Inhibition of nuclear factor kappa B signal pathway and downstream PTGS2 expression could inhibit inflammatory response and protect the perihematomal brain tissue in rats.^[48]^ Furthermore, PTGS2/COX2 is recognized as a significant biomarker of ferroptosis,^[48]^ with upregulated PTGS2/COX2 levels being associated with oxidative stress markers such as ROS, malondialdehyde, and glutathione peroxidase. These markers contribute to secondary brain injury.^[49]^ Our KEGG pathway analysis confirms that PTGS2, IL6, and VCAM1 are downstream products of the TNF signaling system (Fig. 9), further implicating them in the aggravation of inflammatory responses. Our molecular docking results suggest that quercetin, luteolin, and kaempferol exhibit strong affinity with PTGS2, indicating their potential to inhibit oxidative stress and inflammation.

HMOX1 is one of the stress-induced enzymes, which can catalyze the degradation of heme to carbon monoxide, iron, and biliverdin.^[50]^ HMOX1 is also a key target in the process of ferroptosis.^[51]^ In the acute stage of ICH, HMOX1 catalyzes excessive heme decomposition, resulting in iron overload, which may directly lead to ferroptosis. This similar mechanism has been confirmed in acute myocardial injury caused by hemolysis.^[52]^ The elevated HMOX1 expression level is consistent with our bioinformatics analysis. It is suggested that the flavonoids combine with HMOX1, downregulate the activity of HMOX1, and may also weaken the oxidative stress in ICH secondary injury.

VEGF is a heparin-binding protein closely associated with brain edema. This association is primarily attributed to the binding of VEGFA with VEGFR2 receptors. VEGFA plays a pivotal role in mediating transendothelial transport and the disruption of interendothelial tight junctions, contributing to the development of cerebral edema during the acute phase of ICH.^[53]^ Additionally, vascular leakage can lead to heightened intracranial pressure and neuroinflammation.^[54]^ The upregulation of VEGF expression in acute traumatic brain injury can also trigger neutrophil infiltration, further elevating ROS levels.^[55]^ Our bioinformatics analysis indicates a 2.5-fold increase in VEGFA expression within the GSE24265 dataset (Table S5, Supplemental Digital Content, http://links.lww.com/MD/L375). The molecular interactions of quercetin and luteolin with VEGFA hold promise for mitigating neuroedema and neuroinflammation.

In this study, we have leveraged network pharmacology to identify potential targets, construct PPI networks, explore potential pathways via enrichment analysis, and investigate the protective mechanisms of Zhenzhu Pills in addressing secondary injury in ICH. Additionally, we have employed molecular docking techniques to elucidate the molecular-level mechanisms underlying the therapeutic effects of Zhenzhu Pills. Our utilization of computer simulation methods has enhanced the efficiency of traditional drug screening for ICH in high-altitude environments, offering valuable insights for clinical applications and further research into the efficacy of Zhenzhu Pills.

## 5. Conclusion

This study shows that employed with network pharmacology and molecular docking analysis, the core components of Zhenzhu Pills (quercetin, luteolin, and kaempferol) may directly affect the protein activity and function of PTGS2, IL6, HMOX1, VEGFA, and VCAM1, inhibiting the pathological processes such as inflammation, edema, and oxidative stress. It is also the well-known treatment targets of ICH secondary injury, mediated by multiple signal pathways, AGE-RAGE signal pathway, TNF signal pathway, and so on. Due to the limitations of chemical and biological calculation methods, the results of this study need to be verified by follow-up experiments to provide more experimental basis for the therapeutic effect of Zhenzhu Pills on ICH in plateau.

## Author contributions

**Conceptualization:** Gang Wu, Zeng Ren, Qingpei Hao, Yu Wong, Duo Zha, Xudong Cao, Ruen Liu

**Data curation:** Gang Wu, Zeng Ren, Qingpei Hao, Yu Wong, Duo Zha

**Formal analysis:** Gang Wu, Zeng Ren, Qingpei Hao, Yu Wong, Duo Zha, Xudong Cao, Ruen Liu

**Investigation:** Gang Wu, Zeng Ren, Xudong Cao, Ruen Liu

**Methodology:** Zeng Ren, Qingpei Hao, Xudong Cao, Ruen Liu

**Project administration:** Ruen Liu

**Resources:** Gang Wu, Zeng Ren, Duo Zha, Ruen Liu

**Software:** Gang Wu, Duo Zha

**Supervision:** Ruen Liu

**Writing – original draft:** Gang Wu

**Writing – review & editing:** Gang Wu, Qingpei Hao, Yu Wong, Xudong Cao, Ruen Liu

## Supplementary Material


















