# Supplementary material for: The mechanism of Zhenzhu Pills treating intracerebral hemorrhage secondary injury based on network pharmacology and molecular docking
Source: Medicine (Baltimore). 2024 Feb 16;103(7):e36837. doi: 10.1097/MD.0000000000036837 (PMC10869077; doi:10.1097/MD.0000000000036837)
Supplement: Supplementary file 2 [file medi-103-e36837-s002.pdf]

S2. Componets & MOLID deduplication

Herbs	count	MOLID	Compounds	Structure
caoguo	5	MOL000073	ent-Epicatechin	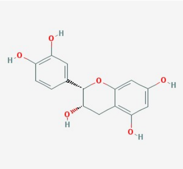
		MOL000074	(4E,6E)-1,7-bis(4-hydroxyphenyl)hepta-4,6-dien-3-one	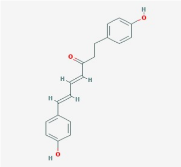
		MOL000085	beta-daucosterol_qt	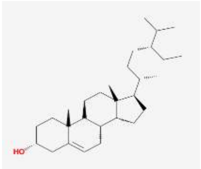
		MOL000096	(-)-catechin	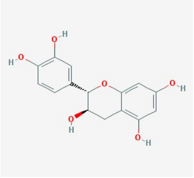
		MOL000098	quercetin	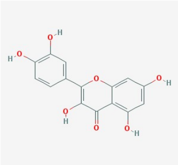
		MOL001006	poriferasta-7,22E-dien-3beta-ol	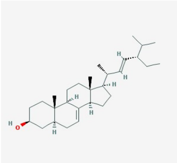
		MOL002140	Perlolyrine	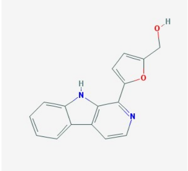
		MOL002879	Diop	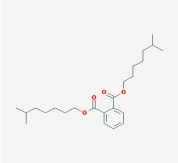
		MOL003036	ZINC03978781	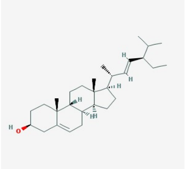

dangshen	15	MOL000449	Stigmasterol	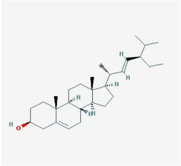
		MOL003896	7-Methoxy-2-methyl isoflavone	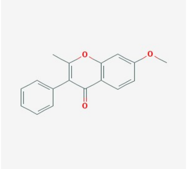
		MOL004355	Spinasterol	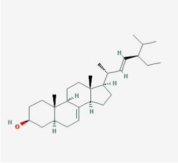
		MOL005321	Frutinone A	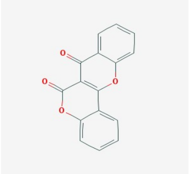
		MOL006774	stigmast-7-enol	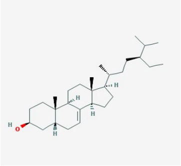
		MOL007059	3-beta-Hydroxymethyllenetanshiquinone	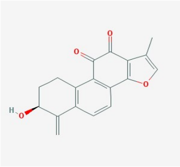
		MOL008397	Daturilin	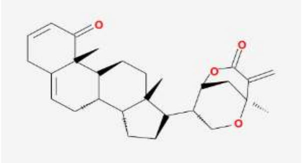
		MOL008400	glycitein	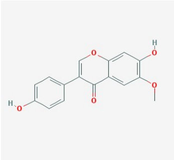
		MOL008407	(8S,9S,10R,13R,14S,17R)-17-[(E,2R,5S)-5-ethyl-6-methylhept-3-en-2-yl]-10,13-dimethyl-1,2,4,7,8,9,11,12,14,15,16,17-	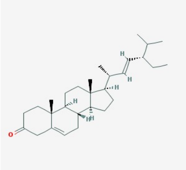
		MOL008411	11-Hydroxyrankinidine	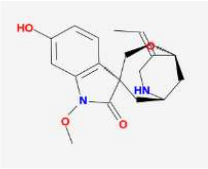

		MOL000006	luteolin	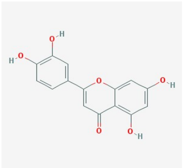
		MOL001040	(2R)-5,7-dihydroxy-2-(4-hydroxyphenyl)chroman-4-one	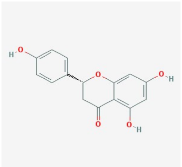
		MOL001484	Inermine	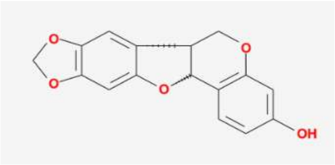
		MOL003542	8-Isopentenyl-kaempferol	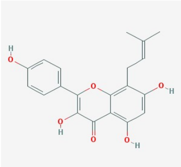
		MOL003627	sophocarpine	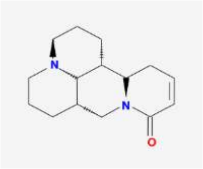
		MOL003648	Inermin	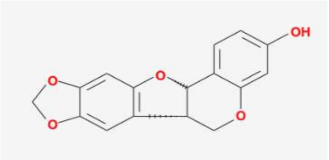
		MOL003673	Wighteone	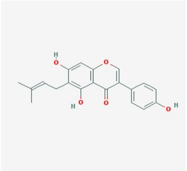
		MOL003680	sophoridine	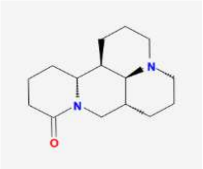
		MOL000392	formononetin	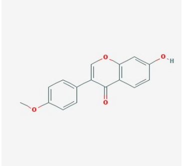
		MOL004580	cis-Dihydroquercetin	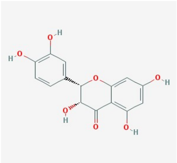

kushen	22	MOL004941	(2R)-7-hydroxy-2-(4-hydroxyphenyl)chroman-4-one	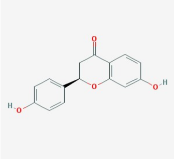
		MOL005100	5,7-dihydroxy-2-(3-hydroxy-4-methoxyphenyl)chroman-4-one	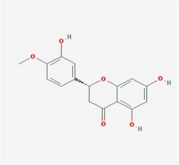
		MOL005944	matrine	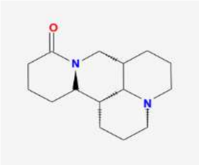
		MOL006596	Glyceollin	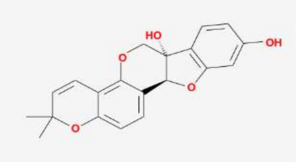
		MOL006604	(2S)-7-hydroxy-2-(4-hydroxyphenyl)-5-methoxy-8-(3-methylbut-2-enyl)chroman-4-one	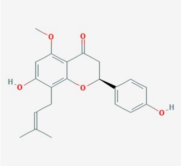
		MOL006613	kushenin	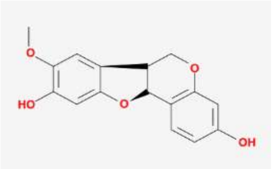
		MOL006620	kushenol J_qt	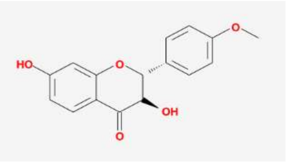
		MOL006623	kushenol,t	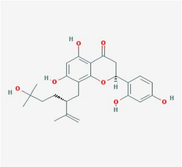
		MOL006626	leachianone,g	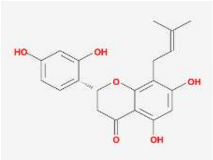
		MOL006630	Norartocarpetin	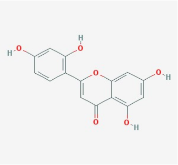

		MOL000456	Phaseolin	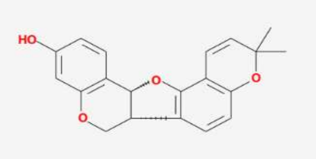
		MOL000098	quercetin	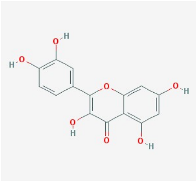
		MOL000006	luteolin	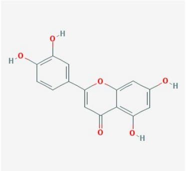
xihonghua	5	MOL001389	n-heptanal	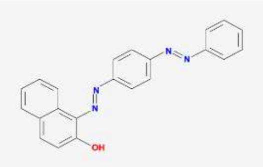
		MOL001406	crocetin	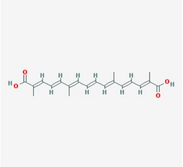
		MOL000354	isorhamnetin	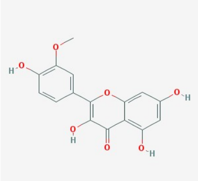
		MOL000422	kaempferol	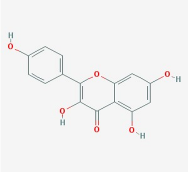
		MOL000098	quercetin	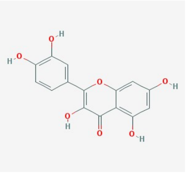
		MOL009295	Flazin	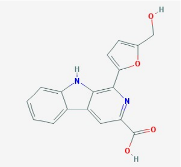
		MOL001939	Alloisoimperatorin	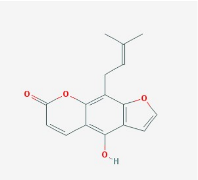

xuelian	7	MOL001735	Dinatin	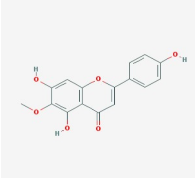
		MOL000358	beta-sitosterol	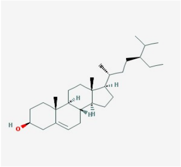
		MOL000006	luteolin	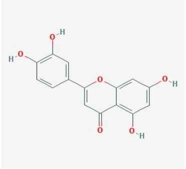
		MOL000422	kaempferol	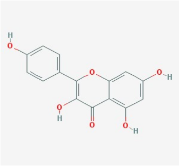
		MOL000098	quercetin	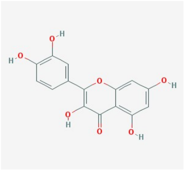
